# Nutritional and Chemical Characterization of Poppy Seeds, Cold-Pressed Oil, and Cake: Poppy Cake as a High-Fibre and High-Protein Ingredient for Novel Food Production

**DOI:** 10.3390/foods11193027

**Published:** 2022-09-29

**Authors:** Diana Melo, Manuel Álvarez-Ortí, Maria Antónia Nunes, Liliana Espírito Santo, Susana Machado, José E. Pardo, Maria Beatriz P. P. Oliveira

**Affiliations:** 1 REQUIMTE/LAQV, Faculty of Pharmacy, University of Porto, Jorge Viterbo Ferreira Street, 4050-313 Porto, Portugal; 2 Higher Technical School of Agricultural and Forestry Engineering, University of Castilla-La Mancha, Campus Universitario, s/n, 02071 Albacete, Spain

**Keywords:** *Papaver somniferum*, by-product, cold-pressing, food security, sustainability

## Abstract

Currently, society demands natural healthy foods with improved nutritional characteristics. Accordingly, poppies (*Papaver somniferum*) are a traditional crop, cultivated for food and pharmaceutical purposes, whose seeds meet consumers’ preferences, making them a promising candidate for incorporation into the formulation of novel functional foods. This work performed an overall chemical characterization of poppy seeds, cold-pressed oil, and press cake, a by-product of the oil industry. The proximate composition, fatty acids, and vitamin E profiles of the oil fraction were analysed with respect to the whole seeds and the cake. The cold-press oil extracted from the poppy seeds was also characterized. Since poppy cake is a partially defatted product, it has a lower fat content than the seeds, but higher content of the rest of its elements, namely, ash (10%), protein (26%), and fibre (38%). Regarding protein composition, the major amino acid in the cake and seeds was determined to be glutamic acid (59 and 36 mg/g, respectively). All the samples presented α- and γ-tocopherols (>21 and >25 mg/kg, respectively) and the fatty acids profile of the oil fraction was mainly composed of unsaturated fatty acids, where linoleic acid predominates (>50%). The oil’s oxidative stability was low (2.8 h), according to the predominance of unsaturated fatty acids. Thus, poppy cake may be considered as an ingredient with great potential for incorporation into products in the food industry according to its high content in protein and fibre, and the remaining fat content, where polyunsaturated fatty acids predominate.

## 1. Introduction

Nowadays, consumers demand functional foods elaborated with new ingredients that provide them with improved nutritional characteristics. They prefer natural ingredients with bioactive compounds, which can promote therapeutic effects. That is why, recently, seeds have attained great popularity. Consumers also prefer minimally processed foods that are obtained sustainably. Since one third of the total food produced is discarded, thereby threatening food security, it is advisable to valorise oilseed-processing by-products to improve food chain sustainability [1,2,3]. In this sense, consumers’ interest in novel foods also includes the wastes generated by the food industry, which may be a source of many healthy compounds whose return to the food chain is within the current guidelines promoted by the European Union with respect to the circular economy and use of waste [4]. In accordance, poppies’ cake is a by-product of the oil extraction industry that still contains many nutritional compounds, which may be useful for incorporation as healthy ingredients in the formulation of functional foods [5].

On the other hand, climate changes’ impact on agriculture, together with the loss of arable land, also reinforce the need for sustainable food production. The rediscovery of ancient seeds provides new alternatives to the food industry and consumers. The search for higher yield species has substituted past crops for more productive ones, threatening biodiversity [6]. Therefore, reintroducing ancient species could solve these problems. Old genotypes, even if less productive than modern ones, are suitable for marginal areas with high-stress conditions. From a nutritional perspective, reintroducing these species in diets can act as a fortifying agent with functional properties and bioactives [6]. Hence, poppy seeds could be further included in the current food market.

Poppies (*Papaver somniferum*) are a major industrial crop cultivated worldwide (Turkey, China, India, Czech Republic) since ancient times for food (oil-rich seeds) and pharmaceutical purposes, i.e., because of opium [7,8,9]. In 2019, the world production of poppy seeds totaled about 30,000 tons of seeds in 56,094 ha, wherein Turkey was the largest producer (27,288 tons; 54,877 ha) [10]. Poppy seeds have different colours (white, yellow, grey, and blue). Their properties and oil yield can vary due to different seeds’ colours and edaphoclimatic conditions [11], although all of them can be used for food purposes such as in oil production or toppings for bakery products. They contain alkaloids with an exceptional medicinal significance (e.g., morphine), and their consumption has been proven to relieve constipation, cough, and asthma [7,8,9]. The seeds contain hardly any opioids but can suffer contamination during harvesting. Opium exposure through poppy seeds represents a serious food contamination problem that can cause, e.g., respiratory depression; thus, control measures are needed to protect consumers [12].

Poppy oil exhibits a great potential for consumption due to its fatty acid profile, where unsaturated fatty acids predominate. Cold-pressed poppy oil is characterized by a nutty smell and flavour [9], which is ideal for use as salad dressing, cooking oil, or therapeutic purposes [13]. To obtain poppy oil, pressure methods are preferred to obtain high-quality oils with a higher presence of minor bioactive compounds such as phytosterols, phospholipids, tocopherols, phenolics, or pigments (such as carotenoids and chlorophylls), as well as other flavor and aroma compounds [14]. On the other hand, poppy cake, which is the by-product of poppies resulting from oil extraction, can be used as cattle feed. However, its protein content makes it a valuable ingredient for introduction in human diets as an alternative to animal protein, providing functional and organoleptic properties to foodstuffs [6,7,9]. In addition, the high nutritional value of this by-product may facilitate its introduction in the food supply chain as a promising novel ingredient. A circular economy, where wastes or other by-products are reintroduced in the human food chain, will benefit society, the economy, and environmental sustainability through a zero-waste approach [1].

Therefore, this work aimed to perform an overall chemical characterization of poppy seeds in their whole form, of their cold-pressed oil, and of the remaining cake as a by-product of the oil extraction industry in order to valorise them, assess their nutritional value, and meet consumers’ food demands (with respect to high-fibre and low-fat foodstuffs).

## 2. Materials and Methods

### 2.1. Raw Materials

Poppy seeds from Czech Republic were purchased in a supermarket (one sample). Oil and cake were obtained by seed pressing (1 kg of the same seeds) in a Komet Oil Press CA59G (IBG Monforts Oekotec GmbH & Co. KG, Monchengladbach, Germany). Oil was submitted to nitrogen stream and stored in an amber container. Seeds and cake were vacuum-sealed. All samples were stored in a refrigerated chamber at 4 °C until analysis. Both the cake and the seeds were milled (GM200 GrindoMix, Retsch, Haan, Germany) to perform the chemical analysis.

### 2.2. Seeds and Cake’s Proximate Composition

Nutritional analysis followed AOAC methods [15]. Moisture content was determined via an infrared balance (DBS—KERN & SOHN GmbH, Balingen, Germany). Total ash and total protein content were determined according to AOAC 920.153 and AOAC 928.08, respectively. The nitrogen conversion factor was 6.25 [16]. Total fat, total dietary fibre (TDF), and insoluble fibre content were analysed according to AOAC 991.36, AOAC 985.29, and AOAC 991.42, respectively. Energy values were estimated according to the following values: fibre (2 kcal/g and 8 kJ/g), carbohydrate/protein (4 kcal/g and 17 kJ/g), and fat (9 kcal/g and 37 kJ/g) [17].

### 2.3. Seeds and Cake’s Total Amino Acids

Total amino acids (AA) were analysed in an integrated HPLC system (Jasco, Tokyo, Japan) consisting of an LC-NetII/ADC hardware interface, two Jasco PU-980 pumps, an AS-4150 RHPLC autosampler, an MD-2015 Plus multiwavelength detector, and an FP-2020 Plus fluorescence detector. Alkaline (potassium hydroxide—KOH 4 M, 4 h for tryptophan) and acid hydrolysis (hydrochloric acid—HCl 6 M, 24 h for the other AA) were performed. Aliquots of neutralized hydrolysates were mixed with L-norvaline (2 mg/mL, internal standard) and the injection conditions followed Machado et al., 2020 [18].

#### Seeds and Cake Protein Quality

The evaluation of protein quality was performed by calculating the amino acid chemical score (AAS) and the essential amino acids index (EAAI), following WHO/FAO/UNU, 2007 [19] and Oser, 1959 [20]:

(1) $$AAS\;\left(\%\right)=\frac{mg\;of\;AA\;in\;1\;g\;test\;protein}{mg\;of\;AA\;in\;1\;g\;requirement\;protein}\times 100$$

(2) $$EAAI\;\left(\%\right)=n^{log\;EAA},where\log EAA=\frac{1}{n}\left(log\frac{100\;a1}{a1R}+\ldots +log\frac{100\;an}{anR}\right)$$

### 2.4. All Samples’ Vitamin E Profile

Seeds and cake lipid fractions were extracted as reported by Melo et al., 2021 [2] using *n*-hexane (HPLC grade) as extracting solvent and 50 µL of tocol (100 μg/mL) as internal standard. Oil was stirred with *n*-hexane (950 μL) and 50 μL of tocol (100 μg/mL) and used for analysis according to the exact conditions described by Melo et al., 2021.

### 2.5. All Samples’ Fatty Acids Profile

Fatty acids (FA) of seeds and cake’s lipid fractions as well as oil (15 mg of oil stirred with 3 mL of *n*-hexane) were derivatized to methyl esters, following ISO 12966-2:2017 [21], and used for analysis. FA profile was assessed in a GC-2010 Plus gas chromatograph (Shimadzu, Tokyo, Japan) according to the exact conditions described by Melo et al., 2021.

### 2.6. Phytochemical Analysis of the Seeds and Cake

The extraction of the phytochemicals of the seeds and cake followed Melo et al., 2021 [2], employing 80/20% methanol/water (*V*/*V*) in agitation (1 h, 40 °C). Oil phytochemicals’ extraction followed Capannesi et al., 2000 [22], employing *n*-hexane and 80/20% methanol/water (*V*/*V*). The total hydroalcoholic solution was used for analysis.

Phytochemical analysis was determined following Costa et al., 2018 [23], in a microplate reader (BioTek Instruments, Synergy HT GENS5, EUA). For total phenolic compounds (TPC), 30 μL of extract, 150 µL of Folin–Ciocalteu reagent (1:10), and 120 µL of 7.5% sodium carbonate—Na_2_CO_3_ (m/V)—were mixed and incubated (15 min, 45 °C). After 30 min, absorbance was read at 765 nm. A gallic acid calibration curve was prepared. For total flavonoids content (TFC), 1 mL of extract, 4 mL of deionized water, and 300 μL of 25% sodium nitrite—NaNO_2_—were mixed. After 5 min, 300 μL of 10% aluminium chloride—AlCl_3_—was added. After 1 min, 2 mL of sodium hydroxide—NaOH (1 M)—and 2.5 mL of deionized water were added. Absorbance was read at 510 nm. An epicatechin calibration curve was prepared.

For ferric-reducing antioxidant power (FRAP) assay, 30 μL of extract was mixed with 270 µL of FRAP solution (0.3 M of acetate buffer, 10 mM of TPTZ (,4,6-tri(2-pyridyl)-s-triazine) solution, and 20 mM of ferric chloride—FeCl_3_). The mixture was kept in the dark (30 min at 37 °C). The absorbance was read at 595 nm. A ferrous sulphate calibration curve was prepared. For 2,2-diphenyl-1-picrylhydrazyl radical (DPPH^•^) inhibition assay, 30 μL of extract was mixed with 270 μL of DPPH^•^ solution (6 × 10^−5^ mol/L in ethanol). The decrease in absorption at 525 nm was measured every 2 min to observe the kinetic reactions until reactions’ endpoint at 20 min. A Trolox calibration curve was prepared.

### 2.7. Oil Stability, Colour, and Regulated Quality Parameters

Oil oxidative stability, expressed as oxidation induction time (h), was determined by Rancimat method (model 892, Metrohm Nordic ApS, Glostrup, Denmark): 3.0 ± 0.1 g of oil, 20 L/h, and 120 °C, as reported by Melo et al., 2021 [2].

Oil colour was determined—following NP-937:1987b [24]—at 445, 495, 560, 595, and 625 nm via a UV Spectrophotometer UV-1800 (Shimadzu, Tokyo, Japan).

Oil peroxide value followed NP-904:1987a [25], namely, 0.5 g of sample, 10 mL of chloroform, 15 mL of glacial acetic acid, and 1 mL of saturated potassium iodide, and these ingredients and KI solution were mixed and stored in the dark (5 min). Then, 75 mL of deionized water was added and a titration with sodium thiosulphate (0.01 N) and 1% starch solution was performed.

Oil UV absorbance followed ISO 3656:2002 [26] with reads at 232 and 270 nm for primary and secondary oxidation products, respectively, via a UV Spectrophotometer UV-1800 (Shimadzu, Tokyo, Japan).

### 2.8. Statistical Analysis

All analyses were performed in triplicate (*n* = 3). Independent Samples T-Test was used to assess significant differences between poppy seeds and cake’s results (*p* < 0.05) in IBM SPSS Statistics (v. 26, IBM Corp., Armonk, 241 NY, USA).

## 3. Results and Discussion

### 3.1. Seeds and Cake’s Nutritional Analysis

The nutritional analysis of the poppy seeds and cake is presented in Table 1. The cake presented significantly (*p* < 0.05) higher content of total ash (10% fw), total protein (26% fw), and TDF (38% fw) than the seeds. Regarding the ash content in the cake (10% fw), it represents a higher source of total minerals than the seeds (7% fw). Previous works have analysed the minerals of different varieties of poppy seeds, as follows: Ca (8756.9–10,702.4 ppm), P (9081.4–10,535.7 ppm), K (6012.1–10,535.7 ppm), and Mg (3406.7–3872.1 ppm) [13]. The cake also showed a high protein content (26% fw), so it could be used as a promising alternative to animal protein in plant-based food diets since the poppy cake contains all the essential amino acids (results discussed below). Furthermore, it is gluten-free, making it a dietary option for coeliacs and consumers who avoid gluten for other motivations. The high fibre content, mainly in the cake (38% fw), includes complex carbohydrate polymers fermentable by the gastrointestinal microbiota into short-chain FAs (acetate, propionate, and butyrate), producing energy and other benefits. Thus, the fibre contained in the poppy cake may contribute to a balanced microbiota composition, which is essential for maintaining health [27].

The seeds presented a higher energy value (2090 kJ/100 g dw) due to their higher total fat content (39% fw) in relation to the cake (10% fw), since fat has the highest energetic contribution (1 g = 9 kcal) [17]. The cake is the by-product obtained after cold pressing, where a significant portion of the oil is removed. Thus, the cake represents a partially defatted product, which meets consumers’ preferences for low-fat foods. Several extraction methods can be applied to acquire different oil yields [30]. To obtain virgin oils, only physical methods are allowed. Therefore, oils are generally extracted by pressure methods, in which two types of presses can be used: the hydraulic press, where the oil is obtained at room temperature, and the screw press, also known as an oil expeller, which is the preferred method since it leads to a generally higher oil yield. Thus, the cake obtained with the screw press shows a lower fat content.

Regarding nutritional characteristics, poppy cake meets consumers requests for natural, chemical-free, gluten-free, high-protein/fibre, low-fat foods obtained sustainably with minimal processing.

### 3.2. Seeds and Cake’s Total AA

The cake contained a higher total AA content than the seeds (274 and 172 mg/g, respectively, Table 1). The major AA identified in both samples were glutamic acid (Glu, 59 and 36 mg/g), followed by arginine (Arg, 32 and 20 mg/g), and aspartic acid (Asp, 27 and 17 mg/g, respectively). Glu is the most common neurotransmitter in the nervous system, stimulating brain function and mental activity. It works as a building block of AA for muscular proteins [2,35]. Arg is responsible for nitric oxide (vasodilator) production and is a precursor of urea, ornithine, and agmatine. In large quantities, it stimulates hormones’ production, e.g., growth hormone and prolactin [2,35]. Asp functions as a brain-excitatory neurotransmitter and participates in gluconeogenesis and the urea cycle. It is a precursor in purines and pyrimidines’ synthesis [2,35].

Leucine, isoleucine, and valine, all branched-chain amino acids (BCAA), are essential AA, since they must be consumed through food, and play several important metabolic functions related to health [2,35]. Poppy cake and seeds are sources of these AA in the following levels: 18 and 11 mg/g for leucine, 10 and 7 mg/g for isoleucine, and 13 and 9 mg/g for valine, respectively. Overall, poppy seeds and cake proteins are dietary sources of all essential AA, including BCAA, amounting to 27 and 41 mg/g, respectively.

When comparing the current results to a previous study [2] on sesame cake and seeds, the total amounts of AA (305 and 199 mg/g, respectively) were higher than in poppy samples. However, the major AA were the same (Glu, Arg, and Asp). In addition, when comparing these results with a study with trending grains in diets, namely, amaranth and quinoa [36], both present lower total protein content (21% and 16%, respectively) than poppy cake (26% fw), but higher content than poppy seeds (15% fw), reinforcing the notion that poppy cake is a better protein and AA source than the seeds. Amaranth and quinoa presented amounts of total AA of 140 and 114 mg/g, respectively [36]. Poppy cake and seeds presented higher amounts of total AA than these grains (274 and 172 mg/g fw, respectively). Unlike the present results, the following AA, glutamine, tryptophan, and lysine, were not identified in either amaranth or quinoa. Furthermore, similarly to sesame, amaranth, and quinoa, poppy protein is a plant-based alternative as well as gluten-free option.

In terms of protein quality (Table 2), the amino acid score (AAS) revealed that the limiting amino acid (LAA—the AA with the lowest AAS) of poppy seeds and cake was tryptophan (114% and 96%, respectively). The EAAI was 133% for the seeds and 117% for the cake, revealing that both products present high-quality protein (since the EAAI > 90%) [35].

### 3.3. Seeds and Cake’s Vitamin E Profile

Vitamin E is composed of eight liposoluble isomers with potent antioxidant capacity (α-, β-, γ-, and δ-tocopherols and tocotrienols). Its main function is the protection of polyunsaturated fatty acids (PUFA) against peroxidation. Its activity may depend on the total tocopherols/PUFA ratio. The presence of vitamin E isomers can extend the shelf-life of foods since they scavenge reactive oxygen species and delay oxidation processes. Moreover, the antioxidant activity of vitamin E isomers protect against oxidative damage, which may help in the prevention of chronic diseases [37]. Regarding the different isomers, it has been described that α-tocopherol is the only isomer incorporated in very low-density lipoproteins, leading to greater biological activity. However, recent studies have demonstrated that γ-tocopherol catches more electrophiles and reactive nitrogen species in inflammation processes than α-tocopherol [38].

The total vitamin E content in the seeds was significantly higher than in the cake (175 > 47 mg/kg, respectively, *p* < 0.05, Table 1), probably due to the higher fat content. α-Tocopherol, the most biological active isomer, was found in both the seed and cake extracts (79 and 22 mg/kg, respectively), thereby supporting the nutritional importance of consuming these products, if possible, as raw ingredients because temperature might impact this vitamin’s availability. Although the extraction method was different, α- and γ-tocopherols were also identified in cold-pressed poppy oil but in a higher total amount (235 mg/kg, Table 3).

Previous studies identified more vitamin E isomers in poppy seeds, namely, α-tocopherol (14 mg/kg), β-tocopherol (5.3 mg/kg), γ-tocopherol (87 mg/kg), γ-tocotrienol (2.1 mg/kg), and δ-tocotrienol (1.6 mg/kg), but a lower total content (110 mg/kg) [28]. However, different extraction methods were used, thereby impairing direct comparisons. Nevertheless, in the present work, a diode-array detector was also used, lending confidence to the isomer identification process.

### 3.4. Seeds and Cake’s FA Profile

The FA profile of the oil fraction of the poppy seeds and cake was analysed. In both samples, the major FA identified were the same but in different relative percentages (*p* < 0.05, Table 1): linoleic acid (LA, C18:2n6c, 63 > 51% in seeds and cake, respectively), followed by oleic acid (C18:1n9c, 23 > 19% in seeds and cake, respectively), and then palmitic acid (C16:0, 11 < 17% in seeds and cake, respectively). A similar profile was previously described [31]. The oil contained in the cake presented higher concentrations of palmitic and stearic acids, but a lower amount of LA in comparison to the seeds, possessing more total saturated fatty acids (SFA, 29%) and fewer monounsaturated fatty acids (MUFA, 19%) and PUFA (52%) than the seeds (14%, 23%, 64%, respectively). In both samples, the minor components (<1%) were palmitoleic (C16:1), α-linolenic (ALA, C18:3n3), and arachidic (C20:0) acids. As the results are presented in relative percentages, the lower values of the PUFA and MUFA content in the cake compared to the seeds leads to an increase in the other FA considered, namely, SFA such as palmitic and stearic acids. The same phenomenon also occurred in the FA ratios since different % of C18:2n6 and C18:3n3 will yield different n6/n3 ratios.

The high content of PUFA in the poppy oil fraction makes this oil susceptible to oxidation, particularly with an increasing degree of damage during harvesting or storage. In addition, the oil extraction process may contribute to high oxidation levels, with γ-tocopherol consumption and increased production of oxidation products reducing shelf-life and quality [40]. This might explain why the poppy cake presented a lower PUFA content (particularly LA) than the seeds since the cake was exposed to an oxygen attack during oil extraction, while the seeds were only ground just before analysis.

LA has an important role in the formation of prostaglandins, leukotrienes, and thromboxanes. However, it can cause inflammatory, hypertensive, and thrombotic activities if present in excessive quantity. Therefore, a balanced LA/ALA ratio is vital for health [3]. In the case of the poppy samples, their high LA concentrations (51–63%) give rise to high values of the n6/n3 ratio (44–81, Table 1) that must be balanced in the daily food pattern with other foods richer in ALA.

### 3.5. Seeds and Cake’s Phytochemical Analysis

Phenolics have been reported as compounds with high antioxidant capacity, thereby protecting against oxidative damage. From a nutritional approach, obtaining phenolic-rich food formulations may decrease the use of antioxidant additives and improve products’ nutritional profile, thereby facilitating the acquirement of functional foods. Their levels depend on several factors (e.g., crop, soil, plant maturity stage, and light period) [41].

The analysis of the total phenolics in the seeds and cake (Table 1) revealed significant differences (*p* < 0.05). The cake presents a higher TPC than the seeds (107 > 58 mg GAE/100 g fw, respectively). The same was true for the TFC, which measures a sub-group of phenolics (139 > 37 mg ECE/100 g fw, respectively), suggesting that the oil fraction poorly contributes to the number of phenolics since with less oil the cake presented a higher content.

FRAP and DPPH ^•^ inhibition are complementary assays for the determination of antioxidant activity based on the principle of metal reduction and the capacity of some organic molecules to scavenge radicals, respectively [41]. Regarding the antioxidant activity (Table 1), there were significant differences (*p* < 0.05) in the FRAP assay: the cake (6 mmol FSE/100 g fw) presented a higher value than the seeds (4 mmol FSE/100 g fw). However, in the DPPH^•^ inhibition assay, the results were not significantly different between the cake and seeds (58 and 46 mg TE/100 g fw, respectively).

### 3.6. Poppy Cake’s Potential in Sustainable Food Production

Poppy cake is a high-value by-product from oilseed processing and provides new food options. It is a natural, minimally processed ingredient, which has the potential to be included in new products’ development by food manufacturers. This by-product can be incorporated in novel foods as a functional ingredient, e.g., in bakery products, as it is a high-fibre source and a gluten-free, high-quality protein source. In addition, since poppy cake contains a lower energy value, this partially defatted ingredient also contributes to the prevention of diet-related diseases [1,2,6,42]. Moreover, it meets the EU proposal of a “Farm to Fork Strategy” (one of the goals of the “Food 2030” policy) that will connect the sectors related to the European food system for future years. It confers a major role to Food System R&I with respect to enabling the necessary changes for sustainable food systems. Research, innovation, and investment are required to develop and implement impactful and scalable solutions for the global food system [42].

Currently, poppy cake is considered a waste product from the oil extraction industry and is used for animal feed. Therefore, the introduction of poppy cake into the food chain is in accordance with the circular economy, which consists in the use of waste to achieve environmentally sustainable production through a zero-waste approach. The use of novel foods is also in accordance with food security needs. Furthermore, this by-product also promotes nutritional security due to its complete composition with respect to nutrients and bioactive compounds [1,2,6,42].

### 3.7. Poppy Oil Characterization

The characterization of oils is a major concern with respect to guaranteeing their quality, avoiding fraud, and establishing a quality grade, especially when novel oils are being considered [43]. A correct characterization must include the analysis of the fatty acid pattern, the presence of tocopherols and other minor bioactive compounds, and other regulated quality parameters such as the peroxide value or the spectrophotometric indexes K_232 nm_ and K_270 nm_.

Table 3 presents the chemical profile of the cold-pressed poppy oil. The oxidative stability measurement revealed an induction time of 2.8 h, probably a result of its high content in PUFA (63%), mostly LAs (62%, essential FA), which are more susceptible to oxidation than SFA (12%) due to the presence of more double bounds in their structure. Another study also performed this test and reported a higher value (5.56 h) but with slightly different conditions (3 g of oil, 20 L/h air flow rate, and at 110 °C) [28], which makes comparison difficult since higher temperatures cause an acceleration of oxidation and since the induction time is reduced.

The poppy oil FA profile showed high levels of linoleic acid (62%), but also revealed oleic acid (24%), palmitic acid (10%), stearic acid (2%), and linolenic acid (1%), whereas palmitoleic and arachidic acids were only present in trace quantities (<0.2%), which is similar to a previous study [28]. Differing from the present work, another study identified margaric, eicosenoic, and erucic acids in small quantities [31].

From a health perspective, both MUFA and PUFA have been reported to act as strong cholesterol-lowering agents. Considering that poppy oil presented a significant amount of MUFA (24%) and a high content of PUFA (63%), its consumption may improve cholesterol metabolism, reducing the risk of cardiovascular disease. LA (62%), together with ALA (1%), are the precursors of biologically active eicosanoids (e.g., prostaglandin, thromboxane, and leukotrienes). Eicosanoids originating from ALA mostly exhibit anti-inflammatory effects, while eicosanoids of LA have pro-inflammatory effects. Lower quantities of eicosanoids derived from arachidonic acid are necessary and biologically active, but larger amounts aid the production of thrombi and atheroma responsible for inflammation [9]. The analysed oil presented a high n6/n3 ratio (62), so it is advisable to include other ALA-rich foods in the diet to balance this ratio.

Lipid peroxidation, especially in PUFA-rich oils such as poppy oil, can be accelerated by high temperatures and poor storage conditions [44]. In this oil, no primary and secondary oxidation products were formed (K_232_ nm and K_270_ nm, respectively); in addition, the peroxide value was low (2.0 meq O_2_/kg), showing that the oil was well preserved. However, the analyses were carried out immediately after oil extraction, which prevented oxidation. In another study, poppy oil was monitored for 6 months, and during the time of analysis, the conjugate dienes and trienes increased [44]. Thus, it seems important to study the best storage conditions for this oil. The addition of natural antioxidants, e.g., essential oils, can improve the stability and extend the oils’ shelf-life, as proven previously [45].

Phenolics avoid oxidation, reducing the formation of toxic compounds, which preserves oil quality [34]. Although poppy oil presented low concentrations (TPC: 4 mg GAE/100 g; TFC: 2 mg ECE/100 g) and low antioxidant activity (FRAP assay: 76 μmol FSE/100 g; DPPH^•^ inhibition assay: 0.5 mg TE/100 g of oil), it should be noticed that vitamin E is its major protective component. Higher amounts of phenolics were mostly present in the poppy cake, as previously discussed.

Vitamin E content is an important indicator of oil quality and may vary between different types of vegetable oils, which are classified into different grades according to quality indexes (e.g., acid value, peroxide value, and iodine value). The oil grade is related to the refinement degree; for instance, a first-grade oil is less refined than a fourth-grade oil, whose degree of refinement may decrease its vitamin E content [38]. As previously mentioned, the peroxide value (2.0 meq O_2_/kg) for this oil was low. Moreover, this oil is an unrefined product and was obtained in a chemical-free manner by pressing the seeds, which preserves its quality. In the vitamin E profile, the most biologically active isomer (α-tocopherol) was identified (13 mg/kg), as well as γ-tocopherol, which was present in the highest quantity (222 mg/kg), and which is the predominant isomer similar to the results found in the seeds, with a total amount of vitamin E isomers of 235 mg/kg. Recent studies indicate that γ-tocopherol is also a potent health-promoting agent similar to α-tocopherol. It prevents oxidative stress and helps decrease inflammation [9]. A higher quantity (222 mg/kg) of this isomer may contribute to the health-promoting properties of the consumption of poppy oil, especially when consumed raw (e.g., in salad dressing).

Regarding vitamin E isomers, previous studies reported different results. Bozan and Temelli, 2008 [28], found amounts of α-tocopherol 55.3, β-tocopherol 16.7, γ-tocopherol 217.4, γ-tocotrienol 14.7, σ-tocotrienol 5.8, and total vitamin E 309 mg/kg, identifying more isomers and, thus, resulting in a higher total vitamin E content in comparison to the present data. However, different extraction methods were used, which impacts the obtained profile. On the other hand, in the study of Dąbrowski et al., 2020 [30], a similar profile was reported, but with lower α-tocopherol, γ-tocopherol, and total vitamin E content (5.90, 115.7, and 121.6 mg/kg, respectively) in comparison to the present data.

During refinement, up to 99% of carotenoids are removed, but cold-pressed oils do not suffer any purification treatments, functioning as a rich natural source of carotenoids [9,39]. Cold-pressed poppy oil presents a β-carotene content that varies between 1.04–2.32 mg/kg [9]. However, the chlorophyll content is lower—0.17 mg/kg [9].

The most well-known feature of biologically active substances is their antioxidant activity, but cold-pressed poppy oil is a relatively low source of tocopherols (235 mg/kg) when compared to other oils, e.g., cold-pressed sesame oil (484 mg/kg) [2], and a rather poor source of phenolics (4 mg GAE/100 g) and flavonoids (2 mg ECE/100 g). Regarding antioxidant capacity, low levels of DPPH^•^-scavenging activity (16.4%), as in the present findings, have also been reported previously [9].

Overall, due to poppy oil’s probable higher price when commercialized, it seems that it can be restricted to some niche markets as a specialty oil. Nevertheless, it possesses other potential applications (e.g., pharmaceutical, cosmetics, and paints/varnishes industries) [13].

## 4. Conclusions

Poppy seeds, along with their press cake obtained after oil extraction, are products that meet consumers’ nutritional awareness and preference for nutritionally desirable foodstuffs, namely, fibre-rich foods obtained sustainably with minimal processing. The poppy cake showed great potential as a high-fibre and high-protein ingredient for the formulation of nutraceuticals and foodstuffs. The consumption of dietary fibre stimulates the gastrointestinal microbiota, thereby improving health. In addition, plant-based protein can be an alternative in vegetarian food patterns.

Regarding the results, the oil can be consumed, preferentially raw, thereby providing essential linoleic acid and α- and γ-tocopherols. However, due to its expensive price, it can be considered a specialty oil restricted to a niche market.

The seeds combine the characteristics mentioned above and are suitable for consumption whole or can be milled in foodstuffs to expand their reach in the current food market, thereby meeting consumers’ demands for natural, minimally processed products.

Further studies are needed that employ different extracting solvents and methods, evaluate the shelf-life period and best storage conditions, and validate the biological effects and health outcomes.

## Figures and Tables

**Table 1:** Chemical profile of poppy seeds, cake, and seeds’ literature data.

Parameter	Seeds	Cake	Seeds’ Literature Data
Moisture (%)	6.15 ± 0.36 ^b^	8.01 ± 0.13 ^a^	3.50–4.76 [13]; 5.3 [28]; 9.97–11.11 [29]
Ash (% fw)	7.21 ± 0.01 ^b^	10.13 ± 0.13 ^a^	4.92–6.25 [13]; 5.9 [28]
Protein (% fw)	14.62 ± 0.01 ^b^	25.80 ± 0.23 ^a^	11.94–13.58 [13]; 21.6 [28]
Total dietary fibre (% fw)	31.82 ± 0.02 ^b^	37.90 ± 0.19 ^a^	18.3 [28]; 22.63–30.08 [13]
Insoluble Fibre (% fw)	31.70 ± 0.08 ^a^	31.20 ± 0.16 ^a^	
Soluble Fibre (% fw)	0.12 ± 0.02 ^b^	6.70 ± 0.17 ^a^	
Fat (% fw)	38.87 ± 0.04 ^a^	10.45 ± 0.16 ^b^	27.71–33.94 [29]; 30.49 [30]; 32.43–45.52 [13]; 39.5 [31]; 40.6–50.2 [32]; 48.31–52.7 [7]; 49.9 [28]; 49.9–52.4 [11]
Remaining carbohydrates (% fw)	1.33 ± 0.34 ^b^	7.71 ± 0.37 ^a^	
Energy value (kJ/100 g dw)	2090 ^a^	1374 ^b^	
Energy value (kcal/100 g dw)	508 ^a^	332 ^b^	
Ash (% dw)	7.84 ± 0.01 ^b^	11.01 ± 0.14 ^a^	
Protein (% dw)	15.96 ± 0.01 ^b^	28.05 ± 0.25 ^a^	
Total dietary fibre (% dw)	33.91 ± 0.02 ^b^	41.02 ± 0.19 ^a^	
Insoluble fibre (% dw)	33.78 ± 0.08 ^a^	33.91 ± 0.16 ^a^	
Soluble fibre (% dw)	0.13 ± 0.02 ^b^	7.11 ± 0.17 ^a^	
Fat (% dw)	41.42 ± 0.04 ^a^	11.52 ± 0.18 ^b^	
Remaining carbohydrates (% dw)	0.87 ± 0.04 ^b^	8.40 ± 0.41 ^a^	
Amino acids (mg/g fw)			
Aspartic acid	16.74 ± 0.89 ^b^	27.31 ± 0.91 ^a^	
Glutamic acid	36.40 ± 1.42 ^b^	58.58 ± 2.38 ^a^	
Serine	8.34 ± 0.35 ^b^	13.52 ± 0.50 ^a^	
Glutamine	0.57 ± 0.02 ^b^	0.86 ± 0.04 ^a^	
* Histidine	5.80 ± 0.22 ^b^	8.84 ± 0.38 ^a^	
Glycine	8.70 ± 0.45 ^b^	14.32 ± 0.59 ^a^	
* Threonine	6.93 ± 0.26 ^b^	11.15 ± 0.45 ^a^	
Arginine	20.25 ± 0.94 ^b^	31.90 ± 1.10 ^a^	
Alanine	7.86 ± 0.34 ^b^	12.44 ± 0.41 ^a^	
Tyrosine	5.18 ± 0.21 ^b^	8.06 ± 0.40 ^a^	
* Valine	8.53 ± 0.31 ^b^	12.57 ± 0.46 ^a^	
* Methionine	4.39 ± 0.18 ^b^	6.74 ± 0.28 ^a^	
* Tryptophan	1.00 ± 0.01 ^b^	1.48 ± 0.16 ^a^	
* Phenylalanine	6.97 ± 0.32 ^b^	10.52 ± 0.63 ^a^	
* Isoleucine	6.93 ± 0.28 ^b^	10.12 ± 0.44 ^a^	
* Leucine	11.45 ± 0.48 ^b^	17.81 ± 0.80 ^a^	
* Lysine	9.10 ± 0.55 ^b^	15.49 ± 1.12 ^a^	
Hydroxyproline	1.22 ± 0.06 ^b^	2.00 ± 0.05 ^a^	
Proline	6.02 ± 0.46 ^b^	9.92 ± 0.27 ^a^	
∑BCAA	26.90 ± 1.07 ^b^	40.50 ± 1.64 ^a^	
∑Total AA	172.36 ± 7.32 ^b^	273.63 ± 10.71 ^a^	
Vitamin E profile (mg/kg)			
α-Tocopherol	79.31 ± 3.21 ^a^	21.70 ± 0.65 ^b^	14.0 [28]; 23.53–28.84 [29]; 26.8–37.2 [13]
γ-Tocopherol	95.60 ± 3.96 ^a^	25.18 ± 0.11 ^b^	87.0 [28]; 263.7–281.5 [29]
Total vitamin E	174.91 ± 7.12 ^a^	46.88 ± 0.67 ^b^	110 [28]; 348.8–623.1 [13]; 550.39–578.43 [29]
Fatty acids profile (%)			
C16:0 (Palmitic acid)	11.00 ± 0.11 ^b^	17.14 ± 0.15 ^a^	8.1–10.1 [11]; 8.93–10.21 [29]; 12.20 [31]; 12.85–18.70 [13]
C16:1 (Palmitoleic acid)	0.22 ± 0.00 ^a^	0.15 ± 0.02 ^b^	0.1–0.2 [11]; 0.27 [31]; 0.58–0.61 [29]
C17:0 (Margaric acid)	0.06 ± 0.00 ^b^	0.13 ± 0.02 ^a^	0.76 [31]
C18:0 (Stearic acid)	2.49 ± 0.14 ^b^	11.45 ± 0.11 ^a^	2.30 [31]; 2.40–4.30 [13]; 2.85–3.17 [29]
C18:1n9c (Oleic acid)	22.50 ± 0.22 ^a^	18.62 ± 0.06 ^b^	13.11–24.13 [13]; 13.3–23.4 [11]; 16.58–21.41 [29]; 22.19 [31]
C18:2n6c (Linoleic acid)	62.80 ± 0.29 ^a^	50.75 ± 0.34 ^b^	52.60–71.50 [13]; 57.91–64.83 [29]; 59.87 [31]; 63.1–74.3 [11]
C18:3n3 (Linolenic acid)	0.77 ± 0.01 ^b^	1.18 ± 0.16 ^a^	0.16–0.50 [13]; 0.47–0.71 [29]; 0.7–0.8 [11]; 1.30 [31]
C20:0 (Arachidic acid)	0.16 ± 0.00 ^b^	0.58 ± 0.03 ^a^	0.1–0.2 [11]; 0.67 [31]
∑SFA (saturated fatty acids)	13.71 ± 0.12 ^b^	29.30 ± 0.23 ^a^	10.6–12.6 [11]; 13.7 [31]
∑MUFA (monounsaturated fatty acids)	22.72 ± 0.21 ^a^	18.76 ± 0.06 ^b^	13.6–23.7 [11]; 22.9 [31]
∑PUFA (polyunsaturated fatty acids)	63.57 ± 0.30 ^a^	51.93 ± 0.18 ^b^	61.2 [31]; 64.0–75.2 [11]
C18:2n6/C18:3n3	81.46 ± 0.95 ^a^	43.60 ± 6.88 ^b^	46.05 [31]
C18:1n9/C18:2n6	0.36 ± 0.01 ^a^	0.37 ± 0.00 ^a^	0.37 [31]
Phytochemical analysis			
TPC (mg GAE/100 g fw)	57.5 ± 2.5 ^b^	107.4 ± 7.8 ^a^	31.27–33.68 mg GAE/g dw [29]; 33.11–144.98 mg GAE/L [8]; 930 mg/100 g dw [28]; 1133.1 mg GAE/100 g [33]; 1937.7 mg GAE/100 g [34]
TFC (mg ECE/100 g fw)	37.3 ± 5.8 ^b^	138.9 ± 4.2 ^a^	1.17–11.28 mg quercetin equivalents/L [8]; 38.7 mg quercetin equivalents/100 g [33]; 63.27–66.48 mg catechol equivalents/g [29]; 676.3 mg quercetin equivalents/100 g [34]
FRAP (mmol FSE/100 g fw)	3.7 ± 0.3 ^b^	6.1 ± 0.1 ^a^	2.72–31.61 mg TE/L [8]; 835.3 mM FeSO_4_/g [33]
DPPH ^•^ inhibition (mg TE/100 g fw)	46.3 ± 4.8 ^a^	58.1 ± 7.4 ^a^	7.58–11.23% [29]; 18.11–126.29 mg GAE/L [8]; 42.6 μg/mL [33]; 44.0–66.5% [34]

* Essential amino acids. Fw—fresh weight, dw—dry weight, BCAA—branched-chain amino acids, TPC—total phenolic compounds, GAE—gallic acid equivalents, TFC—total flavonoid content, ECE—epicatechin equivalents, FRAP—ferric-reducing antioxidant power, FSE—ferrous sulphate equivalents, DPPH ^•^—2,2-diphenyl-1-picrylhydrazyl radical, and TE—Trolox equivalents. Values represent mean ± standard deviation of triplicates (*n* = 3). Different superscript letters in the same row denote significant differences (*p* < 0.05) by Independent Samples T-Test (IBM SPSS Statistics). Soluble fibre and remaining carbohydrates were calculated by difference [16].

**Table 2:** Composition of amino acids in poppy seeds and cake and comparison in percentage with the requirements of human adults estimated by the World Health Organization (WHO) [19].

EAA	Amino Acid Requirements in Adults (mg/g Protein) [19]	Seeds (mg/g Protein)	Cake (mg/g Protein)	Seeds AAS (%)	Cake AAS (%)
Histidine	15	39.67 ± 1.50 ^b^	34.27 ± 1.47 ^a^	264.48 ± 9.98 ^B^	228.49 ± 9.81 ^A^
Isoleucine	30	47.41 ± 1.91 ^b^	39.24 ± 1.72 ^a^	158.03 ± 6.37 ^B^	130.79 ± 5.75 ^A^
Leucine	59	78.29 ± 3.26 ^b^	69.02 ± 3.12 ^a^	132.70 ± 5.53 ^B^	116.99 ± 5.28 ^A^
Lysine	45	62.25 ± 3.74 ^a^	60.03 ± 4.34 ^a^	138.33 ± 8.30 ^A^	133.41 ± 9.63 ^A^
Methionine	16	30.05 ± 1.25 ^b^	26.13 ± 1.07 ^a^	187.84 ± 7.79 ^B^	163.32 ± 6.72 ^A^
Phenylalanine + Tyrosine	38	83.12 ± 3.61 ^b^	71.98 ± 3.87 ^a^	218.74 ± 9.50 ^B^	189.42 ± 10.20 ^A^
Threonine	23	47.41 ± 1.79 ^b^	43.22 ± 1.75 ^a^	206.12 ± 7.78 ^B^	187.90 ± 7.59 ^A^
Tryptophan	6	6.83 ± 0.07 ^b^	5.74 ± 0.63 ^a^	113.89 ± 1.13 ^B^	95.61 ± 10.43 ^A^
Valine	39	58.31 ± 2.15 ^b^	48.72 ± 1.77 ^a^	149.52 ± 5.52 ^B^	124.92 ± 4.54 ^A^
LAA (%)	-	-	-	Trp 113.89 ± 1.13 ^B^	Trp 95.61 ± 10.43 ^A^
EAAI (%)	-	133.40 ± 4.72 ^b^	117.07 ± 5.43 ^a^	-	-

EAA—essential amino acid, AAS—amino acid score, LAA—limiting amino acid, and EAAI—essential amino acid index. Values represent mean ± standard deviation of triplicates (*n* = 3). Different superscript letters in the same row denote significant differences (*p* < 0.05) by Independent Samples T-Test (IBM SPSS Statistics), particularly small letters refer to results in mg/g of protein and capital letters refer to AAS results in %.

**Table 3:** Chemical profile of cold-pressed poppy oil and literature data.

Parameter	Oil	Literature Data
Oxidative stability (h)	2.82 ± 0.02	3.0–9.2 [32]; 5.56 [28]; 5.59 [30]
Colour (x, y)	(0.3794, 0.3673)	
Transparency (%)	52.0	
Dominant wavelength (nm)	581.7	
Purity	32.2	
K_232 nm_	0.024 ± 0.002	
K_270 nm_	0.007 ± 0.001	
Peroxide value (meq O_2_/kg)	1.95 ± 0.04	0.1 [30]; 1.03–1.27 [29]
Vitamin E profile (mg/kg)		
α-Tocopherol	12.79 ± 1.17	5.90 [30]; 19 [39]; 21 [40]; 21.99–45.83 [7]; 55.3 [28]
γ-Tocopherol	222.30 ± 7.37	115.7 [30]; 157 [39]; 195.37–280.85 [7]; 217.4 [28]; 263 [40]
Total vitamin E	235.10 ± 8.53	121.6 [30]; 182 [39]; 284 [40]; 309.4 [28]
Fatty acids profile (%)		
C16:0 (Palmitic acid)	10.15 ± 0.15	7.67–9.91 [7]; 8.5 [39]; 9.91 [30]; 9.79 [28]; 11.6 [40]
C16:1 (Palmitoleic acid)	0.17 ± 0.06	0.1 [39]; 0.13 [28]; 0.15 [30]; 0.15–0.25 [7]
C18:0 (Stearic acid)	2.05 ± 0.06	1.4 [40]; 1.93 [28]; 2.13 [30]; 2.179–2.55 [7]; 2.4 [39]
C18:1n9c (Oleic acid)	24.08 ± 0.14	11.8 [40]; 11.94 [28]; 14.13–19.28 [7]; 14.4 [39]; 15.83 [30]
C18:2n6c (Linoleic acid)	62.44 ± 0.15	68.76–73.92 [7]; 71.35 [30]; 72.3 [39]; 72.6 [40]; 74.47 [28]
C18:3n3 (Linolenic acid)	1.02 ± 0.17	0.55–0.66 [7]; 0.60 [28]; 0.65 [30]; 0.8 [40]; 0.9 [39]
C20:0 (Arachidic acid)	0.09 ± 0.01	0.1 [39]; 0.10–0.17 [7]
∑SFA (saturated fatty acids)	12.29 ± 0.17	13.0 [40]
∑MUFA (monounsaturated fatty acids)	24.25 ± 0.18	13.6 [40]
∑PUFA (polyunsaturated fatty acids)	63.46 ± 0.11	73.4 [40]
C18:2n6/C18:3n3	62.24 ± 10.52	80.3 [39]; 90.75 [40]; 124.12 [28]
C18:1n9/C18:2n6	0.39 ± 0.00	0.16 [28]; 0.19 [39]
Phytochemicals analysis		
TPC (mg GAE/100 g)	3.6 ± 0.4	48.5 mg GAE/100 g [34]; 368.2 mg/L GAE [39]
TFC (mg ECE/100 g)	2.1 ± 0.2	63.27–66.48 mg catechol equivalents/g [29]
FRAP (μmol FSE/100 g)	76.2 ± 9.7	
DPPH ^•^ inhibition (mg TE/100 g)	0.49 ± 0.04	37.2–60.5% [34]; 56.5 mg Trolox/L [39]; 792.6 mg α-tocopherol/L [39]

K—extinction coefficient, TPC—total phenolic compounds, GAE—gallic acid equivalents, TFC—total flavonoid content, ECE—epicatechin equivalents, FRAP—ferric-reducing antioxidant power, FSE—ferrous sulfate equivalents, DPPH^•^—2,2-diphenyl-1-picrylhydrazyl radical, and TE—Trolox equivalents. Values represent mean ± standard deviation of triplicates (*n* = 3).

## Data Availability

Data is contained within the article.
